# Highlight Induced Transcriptional Priming against a Subsequent Drought Stress in *Arabidopsis thaliana*

**DOI:** 10.3390/ijms24076608

**Published:** 2023-04-01

**Authors:** Soyanni Holness, Ulrike Bechtold, Phillip Mullineaux, Giovanna Serino, Paola Vittorioso

**Affiliations:** 1 Department of Biology and Biotechnology ‘Charles Darwin’, Sapienza University of Rome, 00185 Rome, Italy; 2 Department of Biosciences, Durham University, Durham DH1 3LE, UK; 3 School of Life Sciences, University of Essex, Colchester CO4 3SQ, UK

**Keywords:** *Arabidopsis*, priming, highlight, drought, abscisic acid, H3K4 trimethylation

## Abstract

In plants, priming allows a more rapid and robust response to recurring stresses. However, while the nature of plant response to a single stress can affect the subsequent response to the same stress has been deeply studied, considerably less is known on how the priming effect due to one stress can help plants cope with subsequent different stresses, a situation that can be found in natural ecosystems. Here, we investigate the potential priming effects in *Arabidopsis* plants subjected to a high light (HL) stress followed by a drought (D) stress. The cross-stress tolerance was assessed at the physiological and molecular levels. Our data demonstrated that HL mediated transcriptional priming on the expression of specific stress response genes. Furthermore, this priming effect involves both ABA-dependent and ABA-independent responses, as also supported by reduced expression of these genes in the *aba1–3* mutant compared to the wild type. We have also assessed several physiological parameters with the aim of seeing if gene expression coincides with any physiological changes. Overall, the results from the physiological measurements suggested that these physiological processes did not experience metabolic changes in response to the stresses. In addition, we show that the H3K4me3 epigenetic mark could be a good candidate as an epigenetic mark in priming response. Overall, our results help to elucidate how HL-mediated priming can limit D-stress and enhance plant responses to stress.

## 1. Introduction

Any circumstance that can affect plant fitness and significantly reduce yield is referred to as stress. Abiotic stress is managed by plants by either avoiding it or adapting to it [1,2]. Plants have evolved a variety of defense strategies against adverse abiotic conditions because of their sessile nature [3,4]. The plethora of protection mechanisms that plants have developed enables them to not only survive single stresses but a combination of stresses as well. A sophisticated defense strategy developed by plants to combat stresses is known as ‘priming’. Great efforts have been placed into studying this mechanism in recent years due to its importance in crop improvement [5,6]. Although progress has been made in defining and understanding how priming works, there are still a lot of unanswered questions about this phenomenon. Most studies that focus on priming explored the response from using a single type of stress and, to a very limited extent, different types of stress [7,8,9,10,11,12]. In nature, plants are exposed to more than one stress at a time or at specific times. Therefore, it is very crucial to understand this strategy of defense in the context of different stresses.

Drought (D) and highlight (HL) are two abiotic stresses that affect crop productivity. In fact, D is one of the most common abiotic stresses that affect plants’ metabolism, development, and growth. D can be chronic in climates where there is little or no water available, or it can be unpredictable due to fluctuation in weather patterns. Accordingly, it is considered one of the main environmental stresses for plants, especially in areas prone to water scarcity [13,14]. Under D stress, plants detect changes in the soil water deficit and transmit this change from the root to the shoot through mobile signaling to adapt to the changes. For example, the water deficit signal when arriving at the shoot will accumulate abscisic acid (ABA) as an adaptive response. However, ABA-dependent and ABA-independent regulatory mechanisms control the stress response induced by D [15]. For plants that live a photoautotrophic lifestyle, light is the key environmental component because it is the source of energy for photosynthesis and is essential for several plant developmental processes [16,17]. However, when light intensity exceeds the optimal range for photosynthesis, it can cause HL stress in plants. This can lead to the generation of biologically damaging molecules, including reduced and excited oxygen species, peroxides, radicals, and triplet state excited pigments, which reduce photosynthesis performance and lowers plant growth and food production [18,19].

The phytohormone abscisic acid (ABA) regulates the adaptive responses of plants to various environmental stresses and diverse physiological and developmental processes. One of the foremost functions of ABA is indeed the regulation of water and osmotic balance in plants. For instance, ABA is abruptly synthesized to regulate the closing of stomata in response to D. Additionally, activated ABA signaling enhances D stress-responsive genes; however, some D stress-responsive genes are independent of ABA [20,21,22]. Few studies have indicated that plants may become acclimated to HL through ABA signaling [16,23]. For instance, in Arabidopsis, HL-exposed plants showed a two-fold increase in ABA levels compared to plants grown under normal light conditions [16]. Additionally, a phenotypic analysis showed that the ABA biosynthesis-defective mutant *nced3nced5* was hypersensitive to HL compared to the wild type [16]. The induction of the ABA pathway upon both stresses suggests that D and HL might share some of the downstream components involved in a molecular network of plant response to these two stresses.

Priming is normally related to enhanced responses following subsequent stress. For example, hyper-induction of transcription leads to a more robust stress response [8]. In Wheat (*Triticum aestivum* L.), an RNA-seq analysis showed that genes activated by D priming contributed to D tolerance when subsequent D stress occurred [7]. Genes involved in ABA biosynthesis and signaling were identified among the upregulated differentially expressed genes (DEGs) that were grouped in plant hormone signaling. For instance, the *NCED1* gene was significantly upregulated by D-priming after 0.5 h compared to controls [7]. HL in the context of priming is barely studied. Most studies on HL are focused on acclimation and use several light wavelengths. Additionally, a lot of these studies do not separate the heat component from the direct HL effect, thus hampering these analyses. Interestingly, stress-sensitive genes are expressed similarly in plants experiencing HL or cold stress for the first time [10]. A previous study investigated how cold-priming can affect HL stress regulation in *Arabidopsis* [5]. The authors reported that cold-priming affects only a very small subset of genes under HL stress. The cold-priming resulted in increased expression of genes regulating metabolism compared to the expression of defense genes against HL. Overall, they found that cold-priming did not have any or had only very little effect on the HL-triggering responses [10].

Among molecular mechanisms underlying priming, histone modifications are of specific interest since they affect the landscape of transcription of stress-defense-related genes that have been substantially conserved throughout evolution [5]. Recent studies in plants have linked H3K4 trimethylation (H3K4me3), H3K4 dimethylation (H3K4me2), H3K9 acetylation (H3K9ac), H3K12 acetylation (H4K12ac), and H3K27 trimethylation (H3K27me3) to priming [8,24,25]. For example, a study on heat stress in Arabidopsis offers a scenario in which temporary binding of the heat-inducible transcription factor HsfA2 (Heat Shock Factor A2) results in prolonged H3K4 trimethylation and, consequently, the preservation of heat stress memory [12].

The aim of this study was to explore priming responses to D stress using HL as the priming stimulus. We tested for this priming response at the molecular and physiological levels to see if gene expression coincides with any physiological changes. In addition, we have analyzed H3K4me3 as a potential priming/memory epigenetic mark in HL and D stress response.

## 2. Results

### 2.1. Experimental Set-Up for Single D and HL Stress Treatment and Recovery

**Figure 1:**
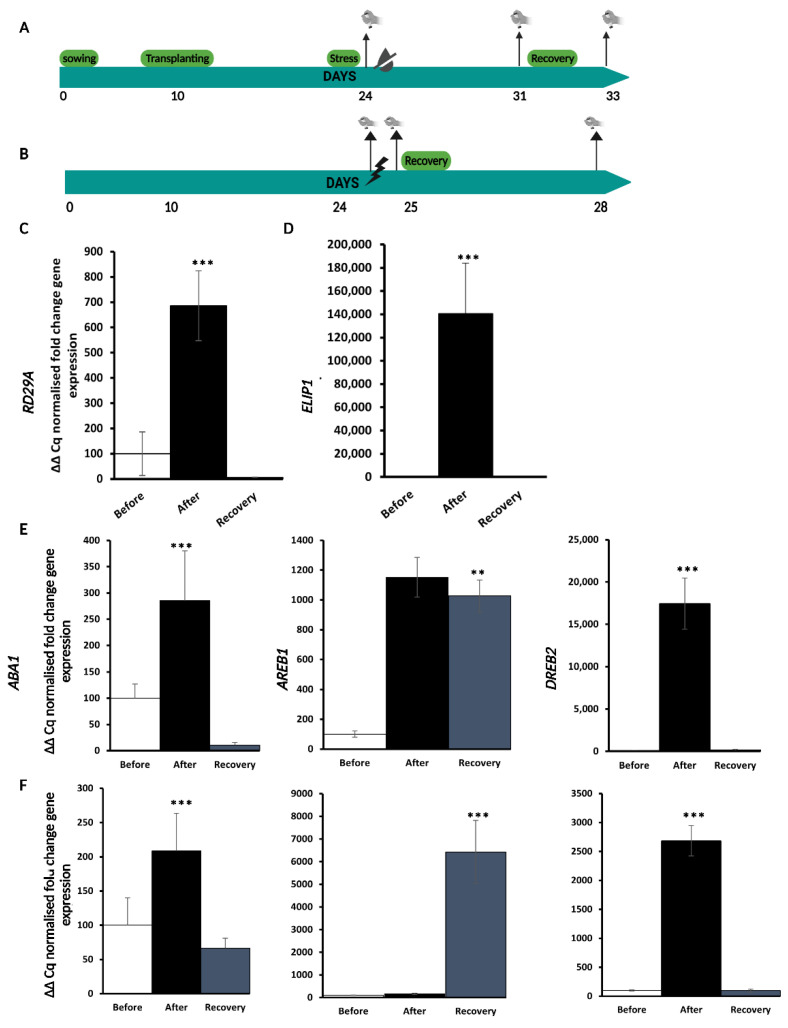
D and HL stress experimental scheme and marker gene expression analysis. (**A**) Experimental scheme for single D stress response and recovery. (**B**) Experimental scheme for single HL stress response and recovery. (**C**) The relative fold change gene expression of *RD29A* at three time points (before = prior to D treatment 24 d, after = immediately after D 31 d, and recovery = 3 days after D treatment ended). (**D**) The relative fold change gene expression of *ELIP1* at three time points (before = prior to HL treatment, after = immediately after HL, and recovery = 3 days after HL treatment ended). Relative expression of ABA genes from single D stress (**E**) or HL (**F**) stress experiment. Data are a representation of one biological replicate with SD values. The two remaining biological replicates are shown in Supplementary Figures S1–S3. Statistical significance analyzed by *t*-test, ** *p* < 0.005, *** *p* < 0.001.

To investigate how the priming effect due to one stress can help plants face subsequent stress of a different type, we decided on HL as a trigger stress and D as the subsequent because D is one of the most frequent abiotic stresses in plants that have experienced an HL stress. To this end, we first performed single-stress experiments to establish a set-up that induces a response to both stresses at a specific timepoint, as outlined in Figure 1A,B. To mimic natural conditions, soil-grown experimental conditions were used with wild-type (WT) Col-0 plants. The experimental set-ups were confirmed by assessing the effects of the stress treatments through the analysis of molecular and physiological markers. These single-stress experiments were conducted on three-week-old plants grown under long-day conditions (16 h of light). We evaluated the effects of D treatment at several timepoints (5, 7, and 10 days) and found that the plants were properly stressed at 7 days and produced the best response, while they were not completely water-deficient at 5 days and on the verge of death at 10 days. Similarly, we evaluated HL treatment at different timepoints (6, 8, and 24 h) and realized that the HL stress response was triggered after 8 h, while plants were not entirely HL-stressed at 6 h and too stressed after 24 h. After stress treatments, the plants were given 3 days of no stress to test if they could recover from the stress that had been induced.

Plants were subjected to D or HL stress as indicated and, to verify the unfolding of a proper stress response, the expression of two different markers, *Responsive to Desiccation 29A*, *RD29A* gene [26] and *Early Light Induced Protein 1*, *ELIP1* [27] for D and HL stress, respectively, were measured at the three time points (before, after, and recovery) as indicated in Figure 1C,D. The two markers were chosen because they have been routinely used in previous HL and D experiments [28,29]. In our experimental set up, *RD29A* expression increased significantly upon D stress and decreased after 3 days of rewatering (recovery), as outlined in Figure 1C. Similarly, to D stress, *ELIP1* expression increased significantly upon HL treatment and decreased after recovery (Figure 1D). This indicates that both D and HL elicit a transient stress response in our experimental conditions.

Given the prominent role of ABA in both D and HL responses [16,23,30,31,32], we checked the expression of a key ABA metabolic gene *ABA Deficient 1*, *ABA1* [33], of an ABA signaling gene *Abscisic Acid-Responsive Element Binding Protein1*, *AREB1* [34] and of a gene *Dehydration-Responsive Element Binding 2*, *DREB2* that is induced by drought independently of ABA [35], to assess if ABA biosynthesis or signaling were differentially regulated upon D or HL stress treatments. These genes were also selected to identify if they can be used as molecular markers to assess the HL effect on D in the sequential stress experiments. Similarly, to the expression of D and HL marker genes, the expression of all three genes increased upon stress, suggesting that ABA response is involved in the plant’s response to these two stresses as shown in Figure 1E,F, and B. *ABA1* expression increased significantly upon D and HL stress and decreased after the stress, similarly to *RD29A* and *ELIP1*, while there were instead significant differences in the expression profiles of *DREB2* and *AREB1* between the two stresses. In fact, *DREB2* expression had a similar profile in both D- and HL-stressed plants, although there was an almost 10x fold increase in *DREB2* expression in D-stressed compared to HL-stressed plants (Figure 1E,F). Conversely, *AREB1* expression followed a different curve in D-stressed plants compared to HL (Figure 1E,F). *AREB1* transcript accumulated mainly upon recovery in D-stressed plants, while it accumulated significantly upon HL stress (10 times increase compared to the same time point on D-stressed plants).

**Figure 2:**
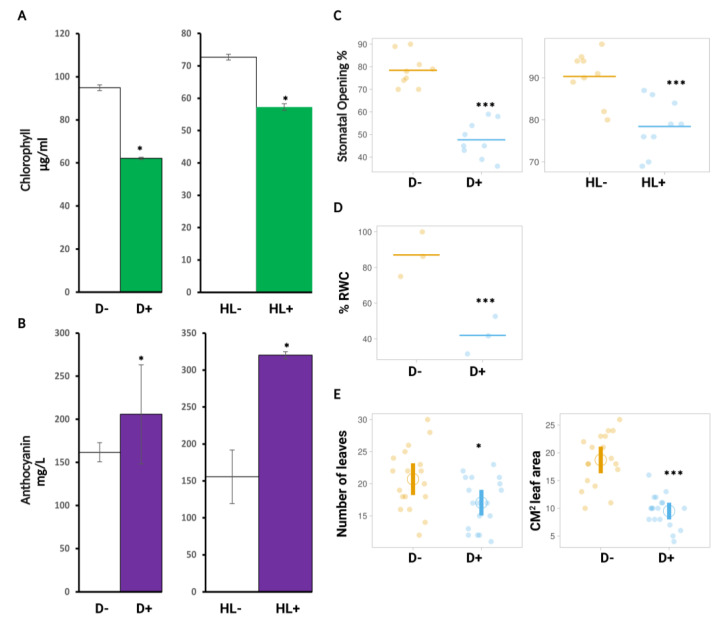
D and HL affect chlorophyll content, anthocyanin, stomatal opening, relative water content, and leaf growth. (**A**) Total chlorophyll from WT leaves after D stress treatment (left) (D- no D stress/control and D+ = D stressed), and after HL stress treatment (right) (HL- = no HL stress/control and HL+ = HL stressed). (**B**) Anthocyanin content from WT after D and HL stress treatment. (**C**) Percentage of stomatal opening in WT after D and HL stress. (**D**) RWC (%) in WT after D stress. (**E**) Total number of leaves and leaf area after D stress measured by image J software 1.52A. Data are a representation of three biological replicates (n = 3) with mean values. Statistical significance analyzed by *t*-test, *** *p* < 0.001, * *p* < 0.05.

Since the photosynthetic apparatus is extremely sensitive to any type of abiotic stress [36], the first physiological marker we assessed was chlorophyll content (a + b). Plants that were not exposed to D or HL stress three-week-old accumulated more chlorophyll in their leaves (Figure 2A). Additionally, stressed plants from the two stress experiments had similar quantities of total chlorophyll compared to the controls. This could suggest that the difference between short-term (HL) stress and gradual (D) stress did not have a noticeable effect on chlorophyll production. The second marker we assessed was anthocyanin content; plants exposed to D or HL stress accumulated more anthocyanin in leaves compared to non-stressed plants (Figure 2B). Noticeably, anthocyanin accumulated significantly more in the HL-stressed plants compared to the D-stressed plants. The controls had similar quantities between both stresses, which further confirms that the plants were indeed controlled. The third marker we assessed was stomata opening. Epidermal peels were created using three or four weeks-old leaves (for D and HL stress, respectively), and the open stomata were counted. When the guard cells were completely or partially open, a stoma was deemed open, and when they were closed, a stoma was regarded as closed. Both D and HL stress significantly decreased the number of open stomata (Figure 2C). We have also assessed relative water content (RWC) and overall plant growth but only for D stress experiments since it is known that these parameters are not primary targets of HL stress [37]. RWC levels after the stress was significantly reduced in stressed leaves compared to non-stressed leaves (Figure 2D), which supports the response observed on stomata opening. As for overall plant growth, we determined it by measuring leaf area and counting the number of leaves. Indeed, leaf area (to a larger extent) and leaf number (to an expected smaller extent) were both significantly decreased in stressed plants compared to non-stressed (Figure 2E). These physiological markers further confirm that the experimental set-up was conducive to establishing both stress responses.

### 2.2. HL Mediates Priming on RD29A and ABA Responsive Genes against D

**Figure 3:**
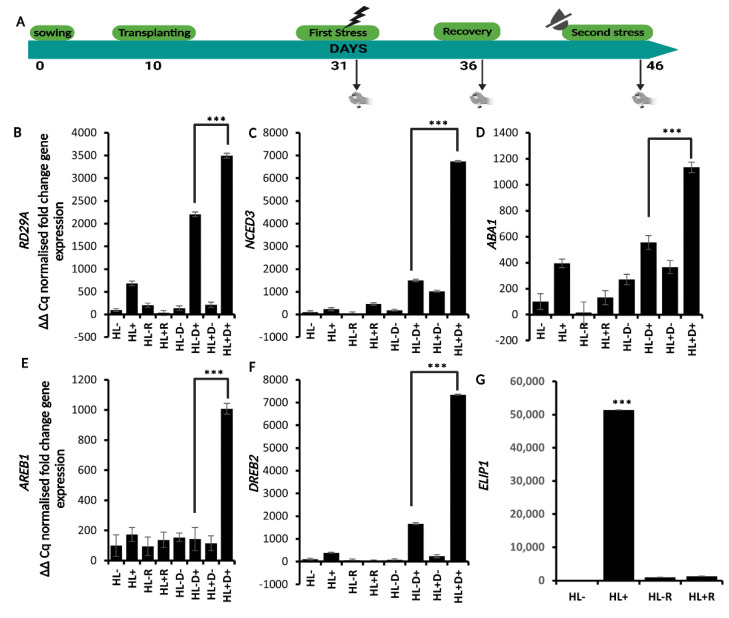
Expression profile of *RD29A-* and other ABA-responsive genes under sequential stress on WT plants. (**A**) Experimental scheme for subsequential HL and D treatment. (**B**–**F**) Expression analysis of *RD29A*, *NCED3*, *ABA1*, *AREB1*, and *DREB2* at 3 timepoints (8 conditions). First timepoint: HL- = no HL stress, HL+ = HL-stressed, Second timepoint: HL-R = no HL stress + recovery, HL+R = recovered HL stress, Third timepoint: HL-D- = no HL and D stress, HL-D+ = no HL only D stress, HL+D- = only HL stress no D, HL+D+ = HL and D stressed. (**G**) Expression analysis of *ELIP1* only at the first two timepoints. Data are a representation of one biological replicate with SD values. The two remaining biological replicates are shown in Supplementary Figures S4 and S5. Statistical significance analyzed by *t*-test, *** *p* < 0.001.

Once verified the effectiveness of the single stress both at the molecular and physiological level, we assessed the effect of HL stress on plant response to a subsequent D stress by setting up a sequential stress experiment. Briefly, plants were treated with HL (priming stimulus), underwent a five-day recovery period, and were then subjected to D stress (triggering stimulus) to identify if the response initiated by HL can prime a tolerance against a subsequent D stress (Figure 3A).

**Figure 4:**
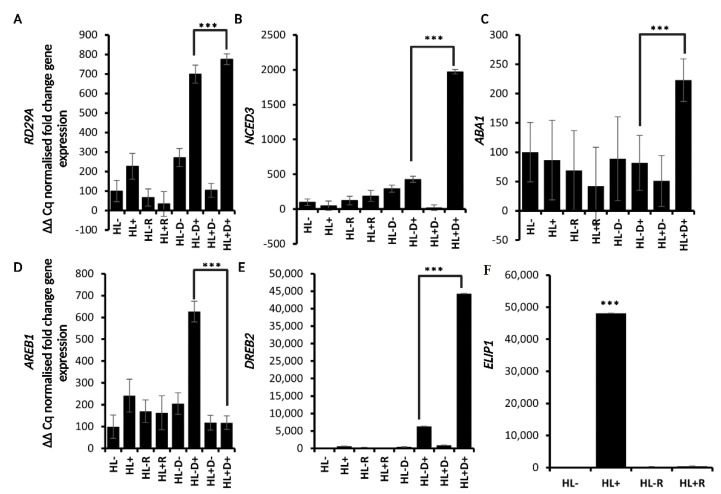
RD29A- and ABA-responsive genes expression pattern in *aba1-3* mutant. (**A**–**E**) Expression analysis of *RD29A*, *NCED3*, *ABA1*, *AREB1*, and *DREB2* at three timepoints. (**F**) Expression analysis of *ELIP1* only at the first two timepoints. Data are a representation of one biological replicate with SD values. Data are a representation of one biological replicate with SD values. The two remaining biological replicates are shown in Supplementary Figures S6 and S7. Statistical significance analyzed by *t*-test, *** *p* < 0.001.

To assess a possible priming effect, the expression profile of *RD29A*, and of the ABA metabolic (*9-CIS-Epoxycarotenoid Dioxygenase 3*, *NCED3,* and *ABA1*), and signaling genes *AREB1* and *DREB2* were evaluated at three time points (HL, recovery, and D) for controls and treatments (Figure 3B–G). Similarly, to what was observed with the single HL stress, *ELIP1* expression (which was only analyzed after HL stress and recovery) was highly induced by the stress, while it dropped down at recovery. Similarly, although to a lower extent, *RD29A* expression increased moderately after HL stress and decreased after recovery. Upon the following D stress, the *RD29A* levels raised again but did not in plants not exposed to D stress. Interestingly, *RD29A* transcript level was significantly higher (about 1.5-fold) in plants that were first exposed to HL and then D if compared to plants only exposed to D stress. *NCED3*, *ABA1*, *AREB1*, and *DREB2* had similar behavior, albeit of different magnitudes (Figure 3B–G). *NCED3* and *DREB2* had the highest peak of expression, followed by *RD29A*, *ABA1*, and *AREB1*. The significant difference in the expression of these genes between plants only exposed to D compared to plants exposed to HL+D suggests that HL stress facilitates the subsequent response to D stress. Moreover, based on the fact that ABA-related genes showed this increased response due to the HL-treatment, ABA might play a role in this priming response. Thus, to gain more insight into the effect of the sequential stress treatment on gene expression and overall plant stress response, the ABA deficient *aba1–3* mutant was also included in our study (Figure 4A–F). Interestingly, the expression pattern of *AREB1* was consistently different in the *aba1-3* mutant compared to the WT, as its expression was higher in D- stressed plants and not in HL+D- stressed plants. Conversely, similar expression patterns were observed between *aba1–3* and WT plants for the other genes, although *DREB2* had a five-fold higher expression in the mutant compared to the WT.

### 2.3. Physiological Markers Assessed for Sequential Stress Experiments

#### 2.3.1. HL Did Not Mediate Priming on Leaf Water Potential, Stomatal Conductance, and Carbon Assimilation in Plants after Subsequent D Stress

**Figure 5:**
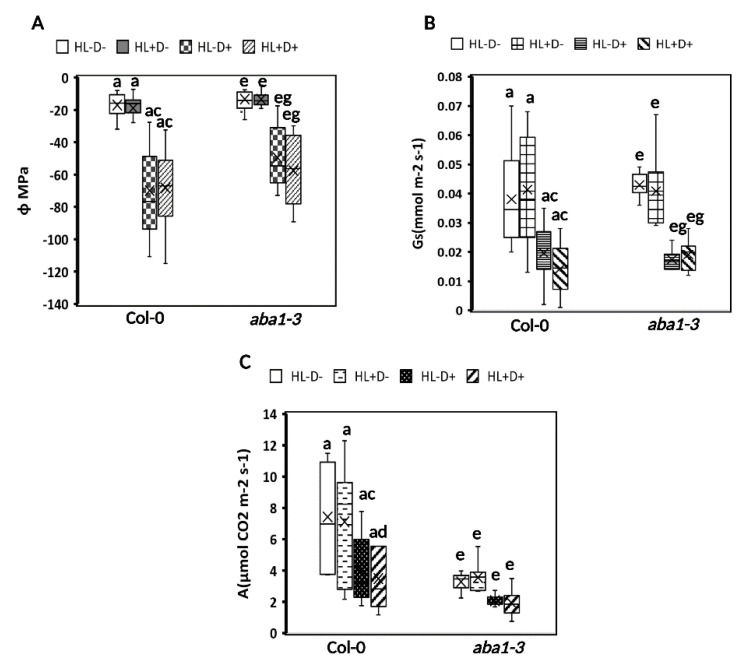
Water, conductance, and assimilation responses were triggered by D, but HL treatment had little to no effect on these responses after subsequent D stress. (**A**) Relative water potential, (**B**) stomatal conductance, and (**C**) carbon assimilation responses were altered by D independent of HL pre-treatments. Measurements were taken at the end of the subjective drought period for all four treatments. The data are a representation of one biological replicate with mean values. The other biological replicates are shown in Supplementary Figure S8. Statistical significance analyzed by one-way ANOVA test and post-hoc Tukey’s HSD test.

Leaf water potential (Φ), stomatal conductance (Gs), and carbon assimilation (*A*) were measured only after the subjected D period to observe if HL triggers any changes in these physiological parameters. Measurements were not taken after HL and recovery to avoid tissue damage, which may impact the recovery and subsequent D treatment (Figure 5). Well-watered plants had higher Φ than D-stressed plants, irrespective of the HL pre-treatment indicating that HL stress did not have a notable impact on the leaf water potential (Figure 5A). There was no significant difference in Φ between WT and *aba1–3* plants suggesting that ABA synthesis is not required for the adjustment of leaf water potential during drought stress. Similarly, stomatal conductance (Gs) was reduced in D-stressed plants compared to well-watered plants in both genotypes, irrespective of the HL pre-treatment. The *aba1–3* mutants had significantly higher stomatal conductance but responded to D treatment with closure (Figure 5B). This suggests that stomatal diffusion was not remarkably affected by previous HL stress and that ABA biosynthesis is not required for a closure response. Carbon assimilation (*A*) followed a similar pattern, with a reduction in D-treated plants, the most significant effect was observed in plants pre-treated with HL (Figure 5C). The *aba1–3* mutants had significantly decreased *A* compared to WT under all conditions, suggesting that ABA synthesis impacts primary carbon metabolism independent of the stress status and stomatal conductance.

Overall, the primary metabolic capacity for these physiological mechanisms did not change significantly between D stressed and HL+D, except for carbon assimilation.

#### 2.3.2. HL and D Did Not Trigger Significant Effect on Photosynthesis Performance and Were Not Targeted for Priming

**Figure 6:**
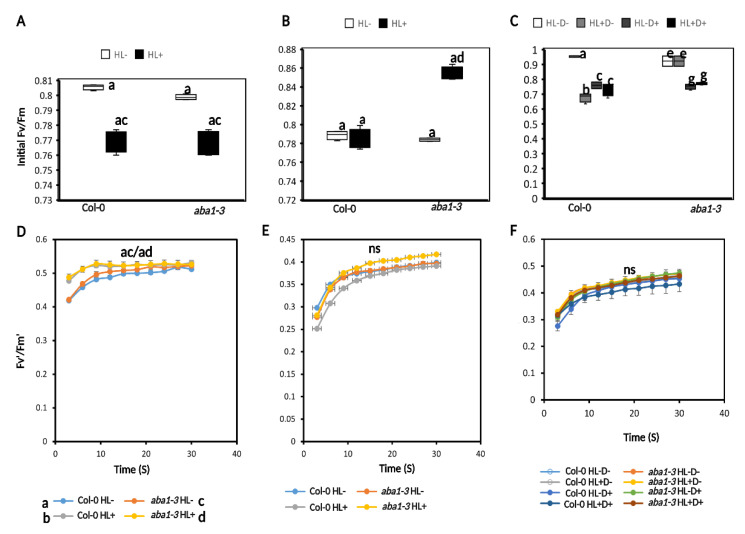
Photosynthetic initiation was not affected by D and HL stress. (**A**) Fv/Fm measurement after HL stress. (**B**) Fv/Fm after 5 days of recovery. (**C**) Fv/Fm after D stress. (**D**) Photosynthesis induction (Fv′/Fm′) over 30 s after HL stress. (**E**) Photosynthesis induction after recovery. (**F**) Photosynthesis induction after D stress. Data are a representation of one biological replicate with mean values. The second biological replicates are shown in Supplementary Figure S9. Statistical significance analyzed by one-way ANOVA test and post-hoc Tukey’s HSD test. ns = not significant.

We measured maximum efficiency of photosystem II under dark- (Fv/Fm) and light-adapted (Fv′/Fm′) conditions using chlorophyll fluorescence (CF) imaging [38]. Measurements were taken immediately after HL, recovery, and D after a 20-min period of dark adaptation. Fv/Fm was reduced after HL exposure for both genotypes compared to controls. The *aba1–3* had higher Fv/Fm compared to WT under low light conditions; however, there were no significant differences between the genotypes after HL exposure (Figure 6A). At recovery, Fv/Fm, there was no significant difference between treatments and genotypes (Figure 6B), which indicates that the plants indeed recovered from the previous HL stress. Subsequent D stress resulted in a significant decrease in Fv/Fm irrespective of the HL pre-treatment in WT. The *aba1–3* mutant had significantly higher Fv/Fm for control and stressed plants after D compared to WT (Figure 6C).

We analyzed Fv′/Fm′ at PPFD 350 µmol m^−2^ s^−1^ for 1 min to initiate photosynthesis induction over 30 s time period. After HL, there was no difference in Fv′/Fm′ between HL- and HL+ plants for WT. However, *aba1–3* HL- plants had a higher induction compared to HL+ plants (Figure 6D). At recovery, no changes were seen between HL- and HL+ for WT but changes were seen for *aba1–3*. The HL+ plants had a higher induction compared to the HL- (Figure 6E). After D, there was only a significant change in induction between HL-D- and HL+D+ for both WT and *aba1–3* (Figure 6F). Interestingly, the mutant had slightly higher overall induction at the three time points compared to the WT.

**Figure 7:**
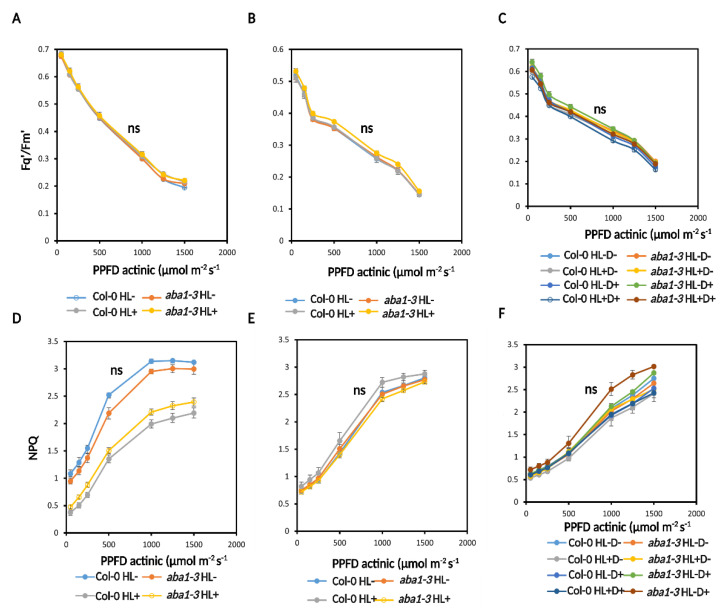
Photosynthesis performance was not affected in response to D and HL. (**A**) Photosynthesis operating efficiency measured by CF imaging using a light response curve of actinic light between 50–1500 µmol m^−2^ s^−1^ after HL stress. (**B**) Photosynthesis operating efficiency measured after recovery stress. (**C**) Photosynthesis operating efficiency measured after D stress. (**D**) NPQ measured using a light response curve of actinic light between 50–1500 µmol m^−2^ s^−1^ after HL stress. (**E**) NPQ measured after recovery. (**F**) NPQ measured after D stress. The second biological replicates are shown in Supplementary Figure S10. Statistical significance analyzed by one-way ANOVA test and post-hoc Tukey’s HSD test. ns = not significant.

We also measured a light response curve ranging from 1500–50 µmol m^−2^ s^−1^ PPFD to quantify the operating efficiency of PSII (Fq′/Fm′) and non-photochemical quenching (NPQ). No changes were seen between treatments in Fq′/Fm′ at HL, recovery, and D for WT and *aba1–3* (Figure 7A–C and Figure S11). WT and *aba1–3* plants had significantly higher NPQ under low light conditions compared to HL-treated plants. After recovery and post-D stress, there was no significant difference between genotypes or treatments (Figure 7D–F).

In conclusion, there was no evidence of a priming effect on photosynthetic parameters. Plants responded to HL treatment with an expected transient reduction in Fv/Fm that recovered post-stress. Drought stress led to a small but significant reduction in Fv/Fm but not the light-adapted parameters (Fv′/Fm′, NPQ, and Fq′/Fm′). Pre-treatment of HL did not impact on subsequent drought stress performance of the plants, irrespective of the ability to increase ABA levels. This suggests HL treatment may not result in physiological priming.

### 2.4. H3K4me3 Was Enriched upon D and HL Stress

**Figure 8:**
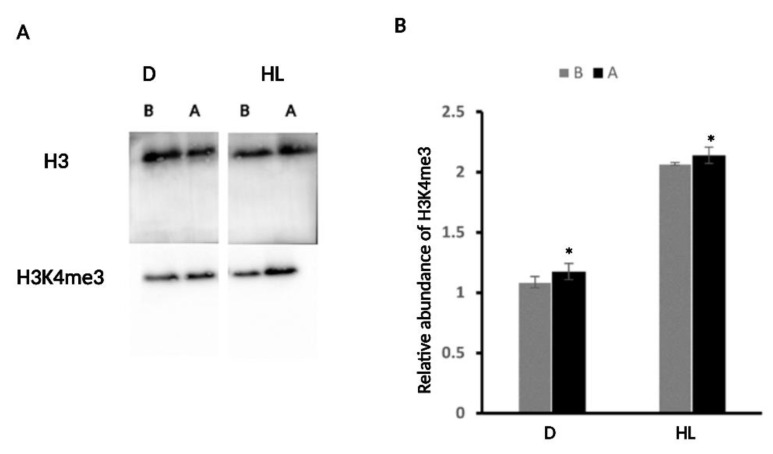
H3K4me3 increased in response to D and HL. (**A**) Immunoblot of WT plants stressed with D and HL at 3 weeks old. B = before stress A = after stress. Histone extracts were probed with H3K4me3-specific antibodies, and H3 was used as loading control. (**B**) Densitometric analysis performed by Bio-Rad Image Lab Software 5.1 and shows the relative expression of H3k4me3 in B and A samples. Relative expressions were obtained from three independent replicates with SD values, but the blot shown is from one replicate. The other two replicates can be found in Supplementary Figure S12. Significant differences were analyzed by *t*-test, * *p* < 0.05.

To identify if there were any changes in chromatin organization, specifically histone modification upon D and HL stress independently, we extracted histones from plant rosettes immediately before (B) and after (A) D and HL stress. H3K4me3 is a good marker of stress memory and gene activation [39,40]; therefore, we selected this histone marker. Immunoblotting analysis of histone extracts collected from before and after D and HL samples separately were assessed from blots with the Bio-Rad Image Lab Software 5.1. The histone mark was increased after both stresses compared to the before samples, thus suggesting that the stresses were the cause of the increased level (Figure 8). The difference in the enrichment of the mark (B versus A) is similar between the two stresses. However, from the HL experiment, the plants seem to yield slightly more H3K4me3 in their samples compared to the D experiment.

## 3. Discussion

Priming, an elegant and adaptive mechanism that plants have evolved to cope with multiple stresses, has been deeply studied in terms of recurring abiotic stress. In this work, we aimed to analyze the effects of short-term HL stress and subsequent long-term D stress. Indeed, under natural conditions, plants that have experienced HL stress may undergo D stress as a consequence. We used D and HL as definable and controllable systems because they both can be applied or removed and measured in a thorough way. Additionally, D and HL have been reported to be linked, and possibly there could be cross-talk between these two stresses. For instance, the *Ascorbate Peroxidase 2* (*APX2*) gene is known to be induced by HL. Overexpression of this gene in the gain-of-function mutant *alx8* results in increased drought tolerance of the mutant. *alx8* also illustrates the intricacy of ABA-dependent and ABA-independent transcriptional networks. Indeed, the *alx8* mutant shows upregulation of some components in both pathways [41].

Our single stress experiments allowed us to define the correct experimental conditions and to validate the molecular markers for both types of stress, *RD29A* and *ELIP1,* for D and HL, respectively. The D stress was defined at 7 days water deficit from molecular analyses of gene expression. In addition to *RD29A*, we selected a number of marker genes that have been previously linked to D-stress response [26,42,43,44]. Since it has been shown that D stress response involves both an ABA-dependent and an ABA-independent pathway [45], we assessed the expression of *AREB1*, an ABA-dependent transcription factor, as well as of *DREB2*, an ABA-independent transcription factor. In addition, we evaluated the expression of the ABA biosynthetic genes *ABA1* and *NCED3,* which are induced by both the ABA-dependent and independent pathways [46,47].

Indeed, *RD29A* increased significantly upon D stress, and so did ABA genes *ABA1* and *AREB1*, and the ABA-independent *DREB2* gene. Surprisingly, the induction of *AREB1* was prolonged up to the recovery phase, while the transcript level of the other genes dropped down to baseline levels, as expected. To substantiate the molecular analyses, we assessed a few physiological markers. D stress results in a reduction of chlorophyll content; this result is consistent with what has already been reported by other studies [48,49]. Conversely, the amount of anthocyanins was increased upon D stress. D and other abiotic stresses are known to cause plants to produce anthocyanins. It is well-recognized that these flavonoid pigments are associated with stress tolerance [50,51]. The other parameters that we assessed—stomatal closure, RWC, and leaf area and number—have already been described as markers of plant response to D stress [52,53,54]. The results we obtained are consistent with a reduced water supply, and decreased transpiration, and leaf growth, thus triggering a response to D stress.

Moreover, the experimental set-up of HL stress has been verified by assessing molecular and physiological parameters. The transcript abundance of *ELIP1* upon the stress indicated a protective response to the excess light. To maintain normal function under light stress, plants normally induce the light stress proteins Elip1 and Elip2 that protect the chloroplast from photodamage, thus preserving photosynthetic efficiency [55]. Damaged chloroplasts can activate retrograde signaling to the nucleus resulting in the upregulation of stress defense genes to mitigate oxidative stress in response to HL. Additionally, ABA was reported to be involved in this HL-response signaling [23]. The increase in ABA gene expression observed in our study supports an ABA involvement in the response to HL. At the physiological level, similarly to D stress, chlorophyll content was decreased while anthocyanin content increased under the stress. It is well known that chlorophyll and anthocyanin are two key pigments associated with HL stress response in plants [56]. Although light triggers stomata opening to promote photosynthesis and photorespiration, stomatal closure in response to HL stress (depending on the intensity of the light stress) is induced to limit transpiration and photoinhibition [57]. Accordingly, our results demonstrated that following HL stress, stomatal opening was reduced to minimize photoinhibition.

Given that our single-stress experiments were able to induce a response, we assessed how HL stress could influence the response of plants to D stress. HL was used as the first stress (priming stimulus), and D was the subsequent stress (triggering) to activate this response. HL was chosen as the first stress because of the nature of the stress; it is instantaneous and can stimulate a rapid response that can quickly be recovered. While D was chosen as the second stress as this stress is more progressive, and damages are not as easily recovered.

We found that after HL treatment, both in HL-stressed and control plants, the transcript levels of *RD29A*, *AREB1*, *NCED3*, *ABA1*, and *DREB2* genes were upregulated in response to the stress. Interestingly, after five days of recovery, the transcript levels of these genes were reduced almost to the basal level suggesting that the HL-stressed plants go back to a ‘normal’ state. The increased transcripts levels of these genes in response to D demonstrated that the plants perceive and respond to the second stress. Interestingly, plants exposed to HL then D (HL+D+) showed a higher expression level compared to plants only exposed to D (HL-D+) or to HL (HL+D-), thus suggesting that HL primes the expression of these genes in response to the D stress. These findings proved that the priming effect on different subsequential stress, as with HL and D, causes a greater upregulation of stress-related genes in primed plants, in agreement with previous data on plants subjected to the same sequential stress, i.e., primed with the same stress [5,58,59].

The involvement of ABA in plant response to abiotic stresses is well established. Based on the gene expression analysis we performed, ABA biosynthesis is induced and targeted for priming. This could suggest an increase in ABA levels, which led to increased expression of downstream ABA-dependent stress response genes through *AREB1*, an ABA-dependent transcription activator. *DREB2* is known to regulate ABA-independent gene expression in response to D. The gene was also upregulated and targeted for priming. There is not a recognized *DREB2*-regulated response to HL stress described so far. This may explain why, even though HL caused *DREB2* expression to be induced, it was expressed at low levels. According to these findings, we suggest that there may be an ABA-dependent and ABA-independent response to the HL-mediated priming on gene expression.

To better understand ABA's role in this HL-mediated priming on these genes, the ABA deficient mutant *aba1-3* was used in our study. Interestingly, the expression profile of the selected marker genes was similar to that of the WT, although the transcript levels were lower, indicating that the expression of these genes was impaired. The only gene whose expression did not exhibit this priming effect was *AREB1*. This data further supports the notion that an ABA-dependent and ABA-independent response might be triggered.

Since HL mediated a priming response on gene expression against D, the next step was to investigate if this trans-priming (priming between two different stresses) is activated at the physiological level. Φ is a good indicator to monitor D stress. This physiological mechanism is reported to be reduced under D stress [60]. Interestingly, the primed plants did not have a change in their leaf water potential in comparison to the unprimed plants. The only difference was between controls (HL-D-, HL+D-) and stressed (HL-D+, HL+D+) plants. It is possible that the plants become adaptable at the D-induced Φ rather than further increasing the water potential capacity as a drought avoidance mechanism. This may imply that if the plants were subjected to D stress for a longer period, the primed plants would be able to endure at the current leaf water potential longer, whereas the unprimed plants may have perished. Gs did not differ between primed and unprimed plants, similar to Φ. Along with temperature, vapor pressure, and ambient CO_2_ concentration, Φ, also known as turgor pressure, influences Gs [61]. Given that there was no change in Φ, it is possible that the other components were also impacted similarly, and as a result, Gs was unaffected. Gs only varied between stressed plants (HL-D+, HL+D+) and controls (HL-D+, HL+D-). *A* rate is reported to be parallel with Gs [62]. The *A* rate showed an interesting pattern, where the primed plants had a lower assimilation rate compared to unprimed plants; however, the unprimed plants had a similar assimilation rate as control (non-stressed plants). *A* rate is the net result of carboxylation and photorespiration [63]. The balance between photorespiration and carboxylation may be proportional to the primed plants’ assimilation rate, or it may simply indicate that one of these processes was operating at a reduced level at the time of measurement. This could be due to damage brought on by the previous HL treatment. The increased assimilation rate in the control and unprimed plants may indicate that the photorespiration and carboxylation processes were more effective since the plants had not previously been exposed to HL. The results from these three parameters suggest a few different situations that might have occurred: 1, no priming memory on the primary metabolic capacity for these physiological systems was mediated by the prior HL treatment; 2, priming might have started, but it was short-lived; therefore, it was not activated by D stress; 3, because the measures were performed after the conclusion of the progressive D treatment, priming may have been engaged at the first feeling of a water deficit, but this information was not captured in our investigation.

Fv/Fm ratios have been largely used as a sensitive metric for plant photosynthetic performance [64]. CF measurements were taken for several Fv/Fm ratios, and the results demonstrated that HL did not mediate a priming response on these parameters against D. The first measurement taken, initial Fv/Fm, was taken at three key time points (HL, recovery, and D) and is a good indicator of the stress status of plants. Plants were indeed stressed after HL treatment as HL+ plants had a lower initial Fv/Fm compared to HL- plants. The plants were able to recover from the HL stress after 5 days, as there was no difference in Fv/Fm between the two groups of plants. The initial Fv/Fm changed again at D, with the stressed plants (primed and unprimed) having a lower Fv/Fm compared to controls (unprimed and non-stressed). These results demonstrated that the plants perceived both stress signals (HL and D) and also unstressed states. The second measurement, Fv′/Fm′, or the induction of photosynthesis, captured the beginning of photosynthesis when the reaction center is open. Most of our results show that HL and D stress had little to no effect on the induction of photosynthesis. The third measurement, Fq′/Fm′, or operational efficiency, was measured using light as the limiting photosynthetic factor; this measurement provides an estimate of PSII operational efficiency. According to much research, this ratio decreases steadily as light intensity rises (mainly light stressors) [65]. After HL and D stress, there was no steady-state decrease in Fq′/Fm′ in our study. In fact, at the three time points (HL, recovery, and D), the photosynthesis rate did not change in plants regardless of treatment in response to the different quantities of light energy. We propose that plants were able to balance photoinhibition and oxidative stress on the photosynthesis machinery by dispersing the effect to their photoprotective channels, which allowed them to maintain effective photosynthesis turnover. The fourth measurement, Fm/Fm′-1 or NPQ, serves as an indicator of the amount of non-reactive energy that PSII dissipates as heat [66]. Most of our results showed no changes in NPQ between stressed and non-stressed plants at HL, recovery, and D. This is consistent with what we suggested, as was stated earlier. The capacity of the NPQ channel was not greater than that of photochemistry but a balance between both. The results from the quantum yield of fluorescence measurement did not show any variation between stressed and non-stressed plants, whether primed or unprimed. The quantum yield of fluorescence is a proxy measure of the energetic efficiency of photosynthesis [67]. The purpose of this measurement was to assess whether the results were comparable to those obtained through CF imaging on photosynthesis performance. In general, HL did not oppose D by mediating a priming response on these photosynthetic parameters. Since the HL and D stress had little to no effect on photosynthetic capability, it suggests that there were no underlying metabolic restrictions. No differences in photosynthetic capability under D stress have been found in other investigations, such as Bechtold et al. (2016) [62]. These findings suggest a few possible outcomes: 1. Photodamage was induced; however, because of the intensity and length of the HL stress, the response to the excess light energy could have been a rapid one, and plants were able to balance out the conversion of this light energy in PSII overtime by becoming acclimated to the HL stress; 2. the HL stress did not induce severe photodamage therefore, plants were able to maintain photochemistry at an efficiency rate and due to this, priming was not triggered; 3. similar to HL, the D stress did not result in significant damage to PSII; this could be due to the operation of a different electron sink [62] under this stress.

Epigenetic mechanisms such as histone modifications are important processes involved in a wide range of biological phenomena. Histone modification has been linked to priming and stress memory in several studies [68,69]. Our study did not specifically investigate stress memory; however, when there is priming, a memory is involved since this enables the plants to store and apply that information later. Since the H3K4me3 epigenetic mark has been shown to be enriched under D-stress conditions and linked to D-stressed upregulated genes [40,68], this histone modification was examined under D and HL stress to determine whether it was linked to both stress responses and whether it may be used as a stress memory mark. H3K4me3 enrichment in D and HL stressed plants compared to controls suggests that this histone mark is responsive to both stresses and could potentially be a target for stress memory. H3K4me3 is known as an active mark for gene expression [69]. It would be interesting to see if this mark is enriched upon the upregulation of the stress response genes targeted for priming in our study. Our results show that the mark is present; however, it is not clear if the modification mark was a rapid response. Does it change gradually in response to the stress? Moreover, how long does this mark remain? Our result only provides H3K4me3 as a candidate epigenetic mark in HL and D priming and stress memory.

The data gathered from this study has several significant implications for priming as it relates to D and HL stress. It demonstrated molecular events involved in priming mediated by HL against D. The data also shows that cross-tolerance is possible between HL and D, and an ABA-dependent and ABA-independent response is likely associated with this priming effect.

## 4. Materials and Methods

### 4.1. Plant Material and Growth Conditions

Arabidopsis thaliana WT (Col-O) was obtained from the European Arabidopsis Stock Center (https://arabidopsis.info, accessed on 11 January 2023). The *aba1–3* mutant line in Col-0 was kindly provided by Dr. Annie Marion-Poll (INRAE centre IJPB de Versailles-Grignon). For single stress experiments, seeds from *Arabidopsis thaliana*, ecotype Columbia and ABA deficient mutant *aba1–3* were sown in pots and stratified for 3 days at 4 °C in the dark on soil media (COMPO, https://www.compo-hobby.it, accessed on 11 January 2023) before individual seedlings were sown onto a soil mix in pots. The pots were transferred in the growth chamber (24 °C/21 °C, 16/8 h day/night, 150 µM/m^−2^ s ^−1^) under cool-white fluorescent tubes. For the sequential stress experiments, wild-type and *aba1-3* seeds were sown in pots and stratified for 3 days at 4 °C in dark soil media (compost: fine grade vermiculite in a ratio of 6:1) before individual seedlings were sown onto a soil mix in pots. The pots were then transferred in the growth chamber with controlled environmental conditions at 60% relative humidity (RH), 23 °C light/dark temperature, and 8 h photoperiod with an irradiance level of 150 μmol/m^−2^ s^−1^.

### 4.2. Stress Experiments

Two experimental set-ups have been used: for the single D stress experiment, after stratification, individual 10-day-old light-grown seedlings were transferred to individual pot cavities (Arasystem, https://arasystem.com, accessed 11 January 2023) filled with the same amount of soil. Two weeks-old individual leaves were randomly flagged and used later to assess molecular and physiological parameters. At 3 weeks, plants were then subjected to D stress by withholding water. The control (non-stressed) plants were kept watered at 3-day intervals. After 7 days of D stress, plants were re-watered for 3 days. For HL stress, the same set-up described for D was used, except that at 3 weeks old, plants were then administered with HL stress at a light intensity of 1000 μmol/m^−2^ s^−1^, white light for 8 h. The control plants were kept at the usual growing light intensity of 150 μmol/m^−2^s^−1^. The HL treatment was set up and controlled by Heliospectra LX60 lamp and system assistant software 2.0.1. After the stress period, plants were then re-watered for 3 days. For the sequential stress experiment, light-grown 10-day-old seedlings were transferred to trays (one seedling to a plug) filled with the same amount of soil in each plug. Pots with only soil were also set up to determine 0% and 100% soil water content. Plants were kept well-watered until 3 weeks old, then HL stress was administered to half the plants at a light intensity of 1000 μmol/m^−2^ s^−1^, white light for 8 h using Isolight 3 (Technologia Ltd, Fourpaths, UK). HL-stressed plants were then returned to the growth chamber containing the other half of the plants and kept under normal growth conditions for 5 days. D stress was then administered to half the plants treated with HL stress and half the plants not treated with HL stress over a time course of approximately 10 days. The D time course ended when the relative soil water content was at 20%. Pots were weighed each day, and the relative soil water content (rSWC) was calculated each day to arrive at 20%. The control plants were maintained under well-watered conditions at ~95% rSWC, as described in Bechtold et al., 2016 [62].

### 4.3. Sampling

RNA extraction: three flagged leaves from three individual plants were collected as one biological replicate to reduce individual variances. Three biological replicates were collected per treatment condition and genotype per experiment. For the single stress experiment, biological replicates were taken at three time points: before stress, immediately after stress, and after recovery. For the sequential stress experiment, biological replicates were also collected at three time points: immediately after HL, after recovery, and after D. Histone extraction: the rosettes from two different plants were collected as one biological replicate, and three biological replicates were collected per treatment condition and genotype. Biological samples were taken from HL and D single experiments. The samples were collected from stressed and non-stressed rosettes after stress treatment. Chlorophyll content: for single stress experiments, three individual leaves randomly selected from three plants collected and weighed to obtain 0.05 g fresh leaves as one biological replicate per treatment and genotype after D and HL stress treatments. Three biological replicates were collected per experiment. Anthocyanin content: for single stress experiments, three individual leaves randomly selected from three plants were collected and weighed to obtain 0.05 g fresh leaves as one biological replicate per treatment and genotype after D and HL stress treatments. Stomatal opening: for single stress experiments, three individual leaves randomly selected from three plants were collected as one biological replicate per treatment and genotype after D and HL stress treatments. Relative leaf water content: for single stress experiments, three individual leaves randomly selected from three plants were collected as one biological replicate per treatment and genotype after D and HL stress treatments. Three biological replicates were assessed per experiment. Leaf growth- for single-stress experiments, plant growth was assessed by counting the number of leaves from selected plants and quantifying areas of selected leaves. Six individual leaves from six plants were pooled as one biological replicate for leaf area measurement. While six individual plants were pooled as one biological replicate for counting the number of leaves. Chlorophyll fluorescence imaging: for sequential stress experiments, four plants were randomly selected for imaging per treatment and genotype. These four plants were imaged after HL stress, recovery, and D stress. Carbon assimilation rate: for sequential stress experiments, six individual leaves randomly selected from six plants were scanned per treatment and genotype only after D stress. Leaf water potential: for sequential stress experiments, six individual leaves randomly selected from six plants were scanned per treatment and genotype. Stomatal conductance and fluorescence measurement: for sequential stress experiments, six individual leaves randomly selected from six plants were measured per treatment and genotype. The measurements were taken only after D stress.

### 4.4. RNA Extraction

Total RNA was extracted from leaves collected and immediately flashed frozen in liquid nitrogen and then stored at −80 °C until further processing. Total RNA was isolated by using Tri Reagent (Merck, Darmstadt, Germany, # T9424). The procedure was carried out according to the manufacturer’s protocol provided by Merck. Total RNA was then quantified using a nanodrop spectrophotometer (Thermo Scientific, Italy) and a nanodrop ND-1000 spectrophotometer (Labtech, Heathfield, UK).

### 4.5. Expression Analysis

Total RNA (1 µg) was reverse transcribed using the PrimeScript^®^ RT reagent kit with gDNA Eraser (TaKaRa; San Jose, CA 95131, USA) according to the manufacturer’s instructions. qPCRs were performed with an SYBR green fluorescence-based assay using the LightCycler^®^ 480 instrument (Roche, Basel, Switzerland). Standard curves were performed using different concentrations of cDNAs and primer sets to determine the primer efficiency and cDNA concentration suitable for use in qPCRs. A total of 1 μL of cDNA was used, along with the specific primers listed in Table S1. Relative expression levels of gene-specific cDNAs were calculated from threshold cycle (Ct) values and normalized to two reference genes, *ACT8* and *UBQ10,* listed in Table S1, and expressed relative to controls.

### 4.6. Histone Extraction

Plant tissues were flash-frozen in liquid nitrogen and stored at −80 °C until further processing. Tissue was ground in liquid nitrogen, and 2 g of ground tissue was used for histone extraction, as described by Ruta et al. 2019 [70]. The ground sample was dissolved in Chromatin Extraction Buffer (0.4 M Sucrose;10 mM Tris HCl pH 8; 5 mM β-mercaptoethanol; 0.1 mM PMSF; 1X protease inhibitor cocktail, Merck, P9599). The nuclei were pelleted at 4000 rpm for 20 min, at 4 °C, and dissolved in 800 μL ddH_2_O. The histones were precipitated with 150 μL of a NaOH/ β-mercaptoethanol solution (138.7 μL 2 N NaOH and 11.25 μL βmercaptoethanol) and then with 55% TCA solution for 15′ in ice. Following centrifugation for 20 min at 14,000 rpm, 4 °C, the pellet was dissolved in HU Buffer (Urea 8 M; SDS 5%; Tris HCl pH 6, 8200 mM; EDTA 0.1 mM; DTT 100 mM; bromophenol blu), boiled for 10′ at 65 °C and stored till further use.

### 4.7. Immunoblot Analysis

Histones were separated on a 12% SDS-polyacrylamide gel using a gel preparation kit according to the instructions of the manufacturer (Bio-Rad, Hercules, CA, USA). The separated histones were blotted on a PVDF Immobilon-P Transfer membrane (Merck, Millipore, Darmstadt, Germany). Detection of histones was performed with specific antibodies against H3K4me3 (Abcam-ab8580, and against histone H3 (orb10805; Biorbyt Ltd, Cambridge, UK) as a nuclear loading control. An anti-rabbit IgG (ASOP 602; Agrisera, Vännäs, Sweden) and an anti-mouse IgG (A9917; Sigma, Granada, Andalucia, Spain) conjugated to peroxidase were used as a secondary antibody, and the signals were detected with the ECL system. Histone bands were analyzed by Bio-Rad Image Lab Software version 5.1 by performing a densitometric analysis to determine the relative expression of the target histone. This analysis was normalized to H3 and used as loading histone control.

### 4.8. Physiological Measurements

#### 4.8.1. Chlorophyll Content

The protocol is as previously described in Lichtenthaler et al., 2001 [71], except that absolute acetone was used for extraction. The Lichtenthaler equation [72] was used to calculate the total chlorophyll (a + b). Briefly, leaves were ground in 3 mL 100% acetone and placed immediately on ice and kept in darkness. The mixture was then centrifuged for 15 min at 3000 rpm, and the supernatant was collected. A final volume of 4 mL was reached by adding acetone, then the color intensity was measured at wavelengths (661.6 and 664.6) for chlorophyll a and b using a spectrophotometer (Ultrospec 3100 pro).

#### 4.8.2. Anthocyanin Content

Anthocyanins were extracted from leaf samples in 2 mL of 1% HCL (*v*/*v*, in methanol). The mixture was left at 4 °C in the dark for 24 h. Two ml of chloroform and 1 mL of distilled water were added to the mixture and blended to separate other pigments from anthocyanins. The upper water–methanol phase collected that contained the anthocyanins, and the absorbance reading was taken for this phase at wavelength 529 using a spectrophotometer (Ultrospec 3100 pro) as previously described in Zheng et al., 2021 [73]. The Murray and Hackett (1991) equation was used to calculate the anthocyanin content [74].

#### 4.8.3. Stomatal Opening

Each leaf surface was coated with nail polish to create an epidermal impression for visualization of the stomata. The dried layer of nail polish was peeled off by using clear tape and stuck on a slide. An optical microscope (Motic BA410) was used to visualize and count the number of stomata at a magnification of 40× by counting the number of different fields across the slide Rothamsted Research Bioimaging, 2017 [75]. Microscopic images of stomata were also captured using a camera (JENOPTI K, Jena, Germany) and processed by ProgRes CapturePro v2.8.8 software. The total stomatal opening was then calculated by deriving the percentage of opened stomata from the total number of stomata.

#### 4.8.4. Relative Leaf Water Content

The protocol for measuring the relative leaf water content (rLWC) is as previously described in Sade et al., 2015 [76]. Ziploc bags (8 cm × 12 cm) were marked and weighed (BW) before other measurements. Leaves were collected at midday for measurements, according to Matin et al., 1989 [77]. Selected expanded and mature leaves were cut with a scalpel leaving a 1 cm long petiole, and put in the marked bags, making sure it was tightly sealed. The weight is taken of bags containing fresh leaves to determine the total fresh weight (TFW). Three mL of 5 mM CaCl was added slowly, then the bags were sealed, and kept at room temperature (RT) in the dark for 8 h. Leaves were then taken out of the bags, placed between two paper towels to absorb excess water, and weighed to determine the turbid weight (TW). The leaves were then transferred to weighed envelops and allowed to dry for 3 days in an oven at 60 °C. The dried leaves were weighed to obtain the dry weight (DW). The relative water content was calculated using the following formula: RWC = (TWF − W) − DW/TW − DW × 100.

#### 4.8.5. Leaf Growth

The leaf area was photographed at a fixed distance between the camera and the leaf along a known scale bar, and photos were imported into the ImageJ, which was used to calculate the areas of leaves. Leaf area was also measured by a grid counting method to confirm the pixel count measurement by ImageJ. This was performed by tracing the shape of the leaf on graph paper. One square on the graph paper represents 1 cm^2^. The area of the leaf was deduced from the following equation: 1 × (number of complete squares) cm^2^ + ½ × (number of half or more than half squares) cm^2^.

#### 4.8.6. Chlorophyll Fluorescence Imaging

Photosynthetic performance was estimated with a Chlorophyll fluorescence (CF) imaging system (Fluorimager; Technologica Ltd, Fourpaths, UK), exposing the plants to actinic PPFD from 1500 to 50 µmol m^−2^ s^−1^ in 200 µmol m^−2^ s^−1^ steps every 5′ as described previously as previously described in Baker et al. 2008 [38]. Plants were transferred from either growth conditions or stress treatment to the dark for at least 20 min, termed dark adapted. If the study required multiple sets of plants tested on the same day, plants remained in dark conditions until ready. Four plants were randomly selected for imaging per treatment and genotype. These four plants were imaged after HL stress, recovery, and D stress. Dark-adapted plants were placed in the fluorimager, and the program was initiated. Whole rosette CF images were collected at each PPFD and processed using software V2 305 (Technologica Ltd, Fourpaths, UK) to collect numerical data for the calculation of Fv/Fm, Fv′/Fm′, Fq′/Fm′, and Fm/Fm′-1 [38,78]. The raw data were exported to Excel to calculate, plot, and analyze statistically the CF parameters. The fluorimager software produces average data of all leaf pixel values.

#### 4.8.7. Snapshot CO_2_/Carbon Assimilation

On-the-spot net CO2 uptake rates were taken from leaves using a gas exchange system (LI-6800; LI-COR). An area of leaves was clamped by the equipment and acclimatized to similar growth conditions. Leaf area temperature was maintained at 22 °C +/− 2 °C, the light intensity of 150 µmol m^−2^ s^−1^, the vapor pressure of 1 kPa, and ambient CO_2_ concentration of 400 µmol as previously described in Bechtold et al., 2016 [62]. Readings were recorded after the gas exchange system was stabilized and before the leaves had a response to new environmental cues [79]. The area of leaf used was calculated using Image J software 1.52A to determine the carbon assimilation (A) rate of that specific area.

#### 4.8.8. Leaf Water Potential

Leaf water potential was measured by using a psychrometer (Dewpoint PotentiaMeter WP4C, Decagon Devices, Pullman, USA), and the measurement was taken only after D stress. Individual leaves were placed in a sealed chamber, allowed to acclimatize to temperature, and stabilized. The relative humidity in the air above the leave samples was measured and used to determine the water potential of the leaves. Measurements were recorded for both controls and treated plants according to the manufacturer’s instructions.

#### 4.8.9. Stomatal Conductance and Fluorescence Measurement

An LI-600 fluorometer/porometer (LI-COR) was used to measure the stomatal conductance (Gws) and the quantum yield of fluorescence (Φ PSII) from leaves at the same time, according to the manufacturer’s instructions. Briefly, leaves were clamped one at a time and allowed to stabilize in the new environment, and readings were taken instantaneously and recorded.

## Data Availability

The data presented in this study are available in Supplementary Material.

## Supplementary Materials

The following supporting information can be downloaded at: https://www.mdpi.com/article/10.3390/ijms24076608/s1.
