# Supplementary material for: Highlight Induced Transcriptional Priming against a Subsequent Drought Stress in Arabidopsis thaliana
Source: Int J Mol Sci. 2023 Apr 1;24(7):6608. doi: 10.3390/ijms24076608 (PMC10095447; doi:10.3390/ijms24076608)
Supplement: Supplementary file 1 [file ijms-24-06608-s001.zip › ijms-2286896-supplementary.pdf]

Supplementary Information

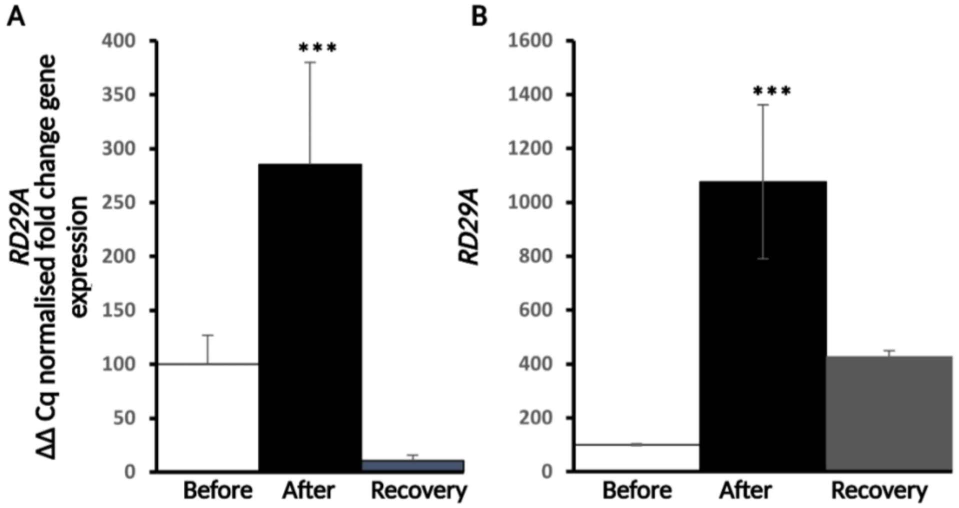

Figure S1: Single D stress experimental scheme and marker gene expression, related to Figure 1
(A) Expression pattern of *RD29A* in WT from second biological replicate. (B) Expression pattern of *RD29A* in WT from third biological replicate. Error bars represent SD values. *** $p < 0.001$.

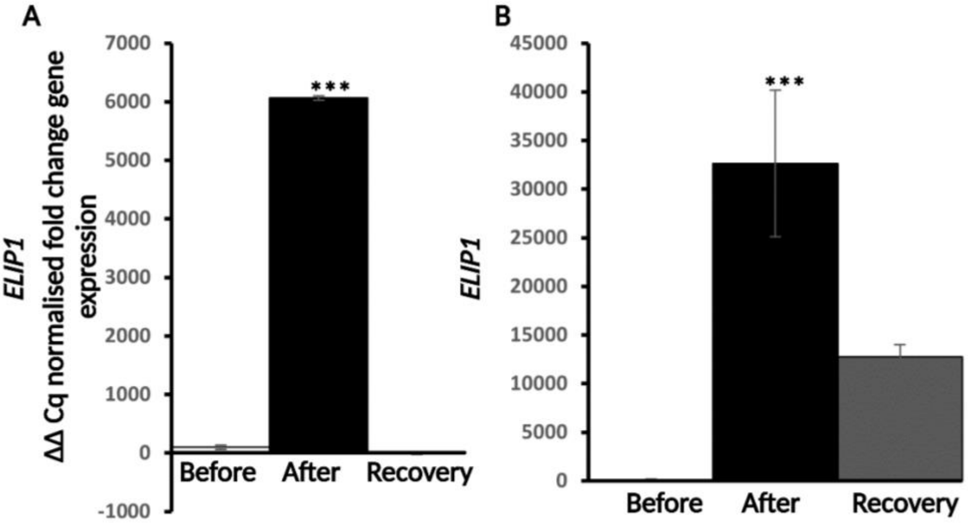

Figure S2: Single HL stress experimental scheme and marker gene expression, related to Figure 2
(A) Expression pattern of *ELIP1* in WT from second biological replicate. (B) Expression pattern of *ELIP1* in WT from third biological replicate. Error bars represent SD values. *** $p < 0.001$.

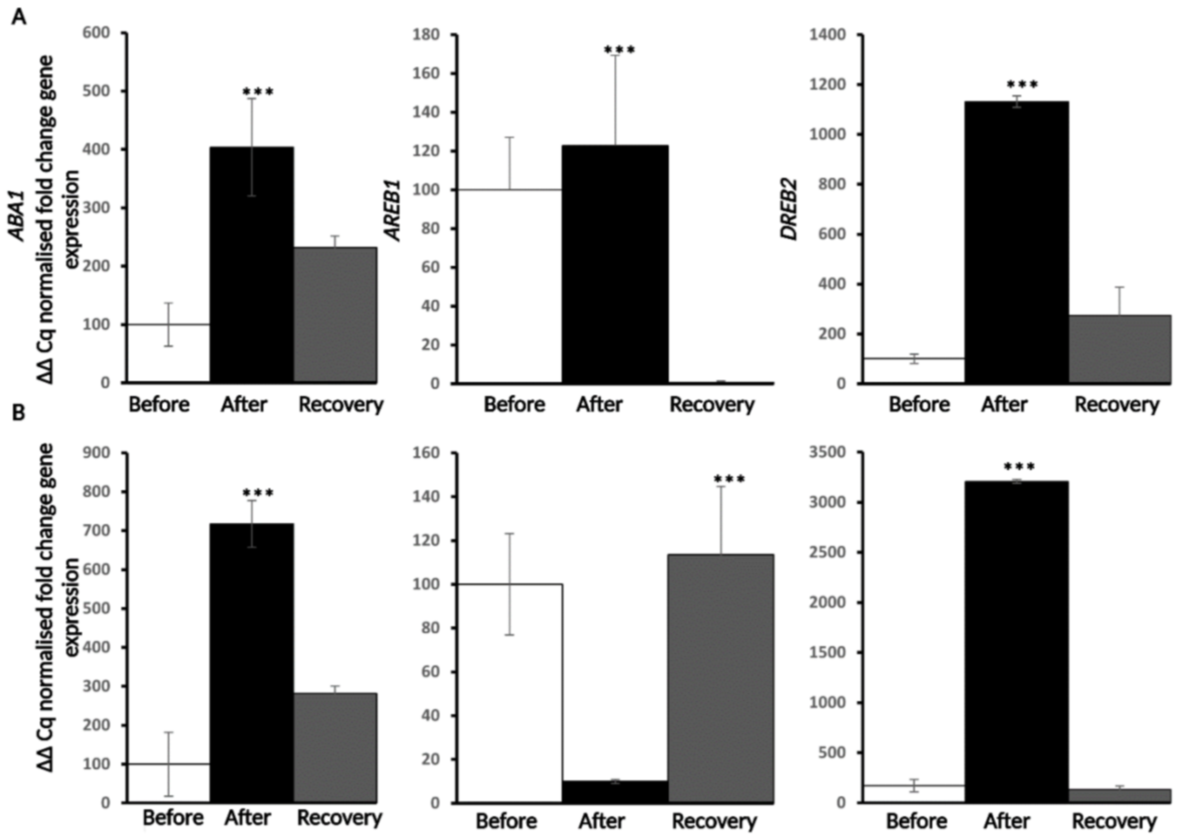

Figure S3. D and HL triggers expression of ABA responsive gene, related to Figure 3

(A) The fold change gene expression of ABA genes in WT from single D stress experiment. (B) Expression of the same ABA genes in WT from single HL stress experiment. Data is a representation of second biological replicate with SD values. *** $p < 0.001$.

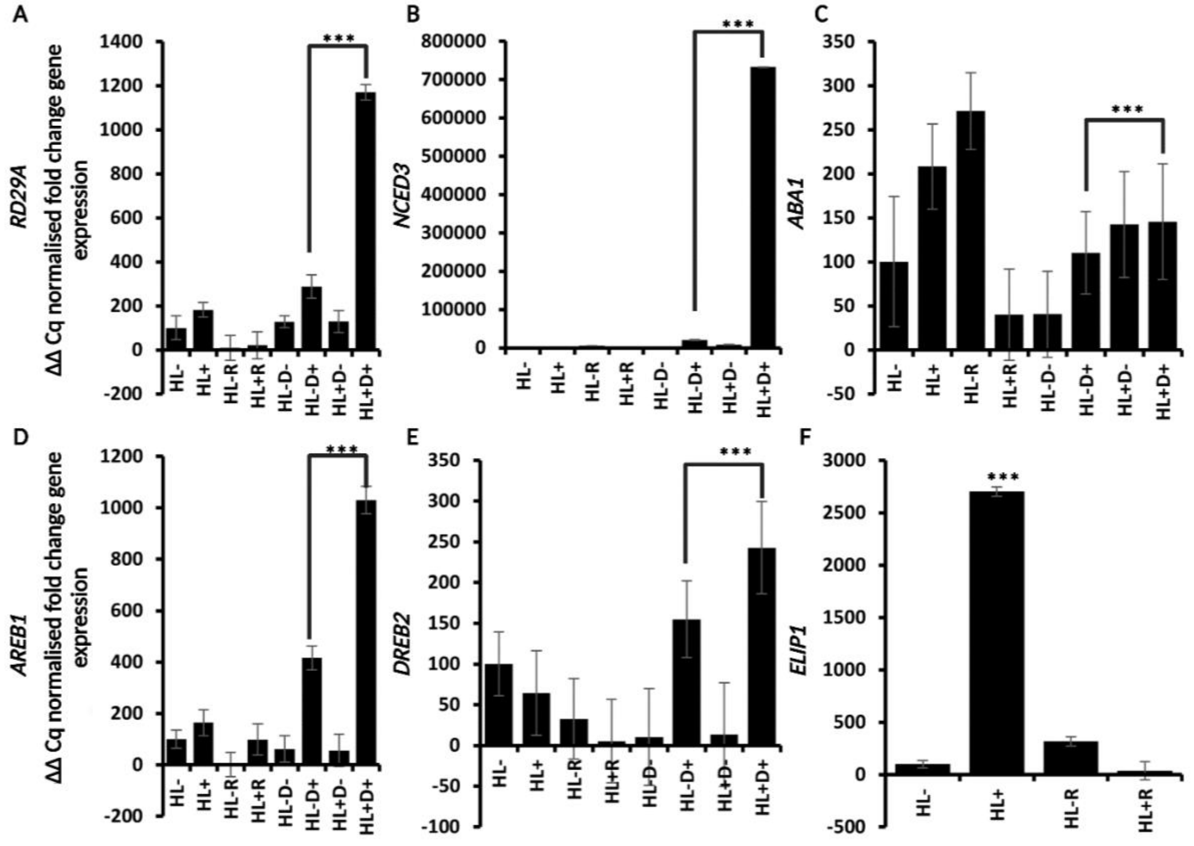

Figure S4: ***RD29A*** **and ABA responsive genes expression pattern in Col-O, related to Figure 5**
(A-E) qPCR analysis of *RD29A*, *NCED3*, *ABA1*, *AREB1*, and *DREB2* at 3 timepoints. (F) Expression analysis of *ELIP1* only at the first two timepoints. Data is a representation of second biological replicate with SD values. *** $p < 0.001$

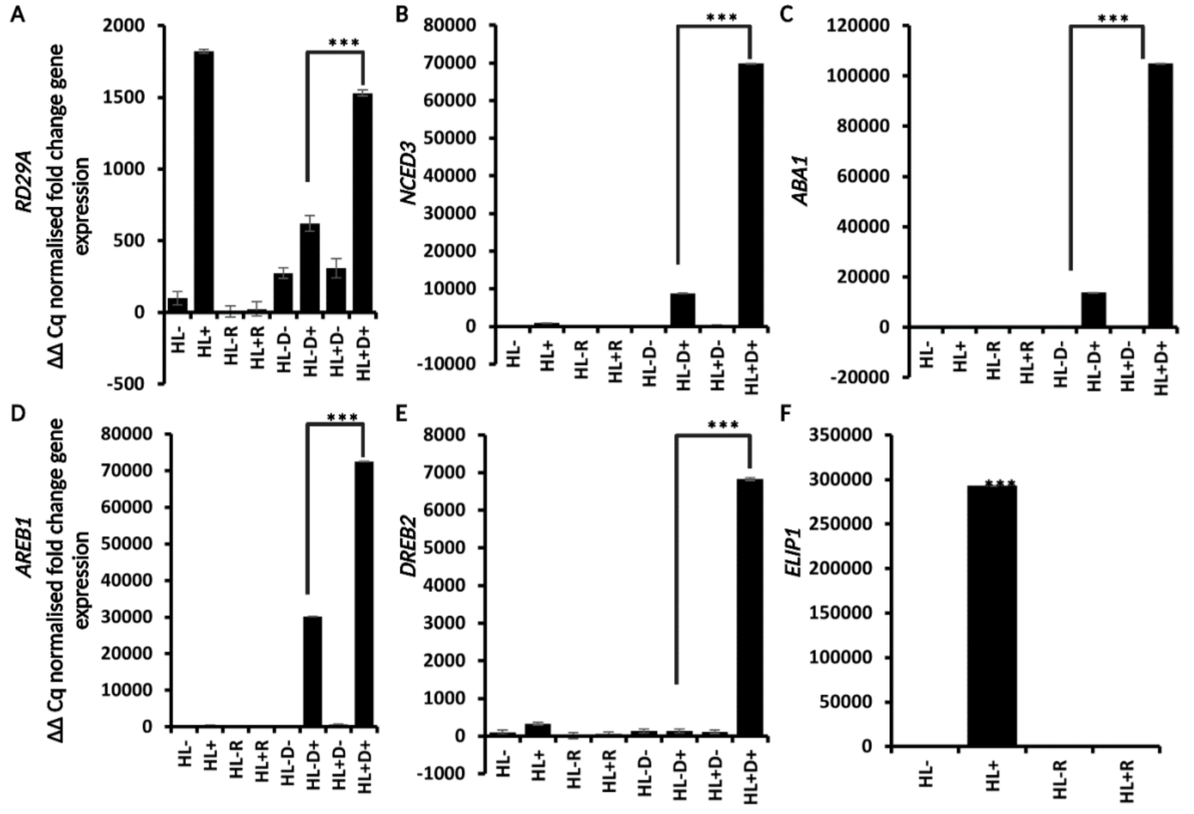

Figure S5: ***RD29A*** **and ABA responsive genes expression pattern in Col-O, related to Figure 5**

(A-E) qPCR analysis of *RD29A*, *NCED3*, *ABA1*, *AREB1*, and *DREB2* at 3 timepoints. (F) Expression analysis of *ELIP1* only at the first two timepoints. Data is a representation of third biological replicate with SD values. *** p < 0.001

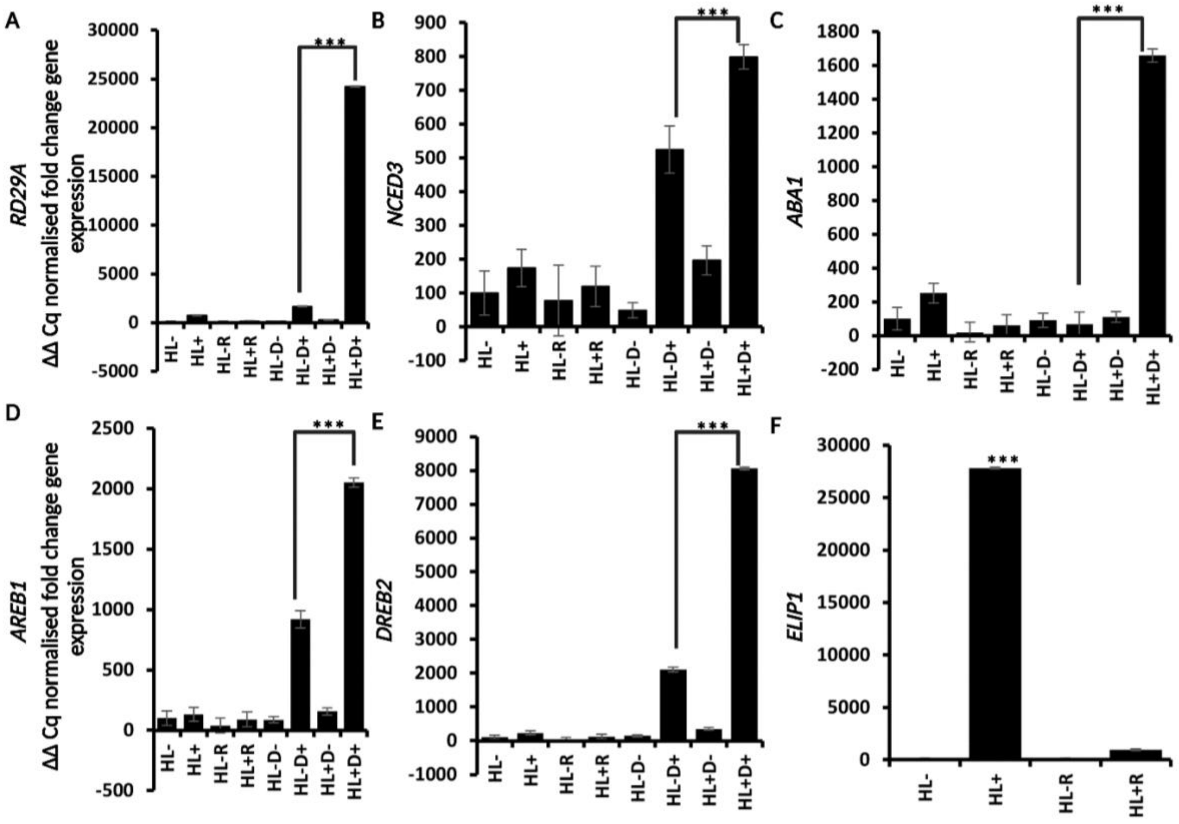

Figure S6: *RD29A* and ABA responsive genes expression pattern in *aba1-3* mutant, related to Figure 6

(A-E) qPCR analysis of *RD29A*, *NCED3*, *ABA1*, *AREB1*, and *DREB2* at 3 timepoints. (F) Expression analysis of *ELIP1* only at the first two timepoints. Data is a representation of second biological replicate with SD values. *** p < 0.001

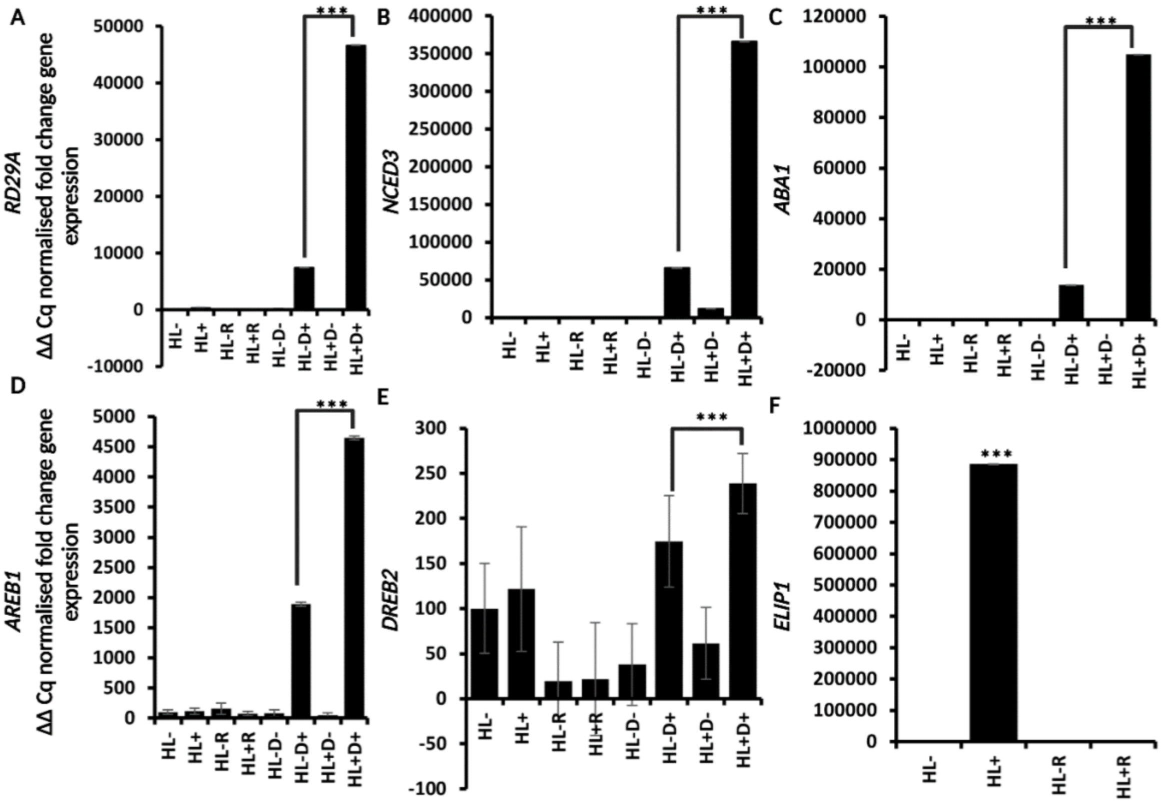

Figure S7: *RD29A* and ABA responsive genes expression pattern in *aba1-3* mutant, related to Figure 6
(A-E) qPCR analysis of *RD29A*, *NCED3*, *ABA1*, *AREB1*, and *DREB2* at 3 timepoints. (F) Expression analysis of *ELIP1* only at the first two timepoints. Data is a representation of third biological replicate with SD values. *** $p < 0.001$

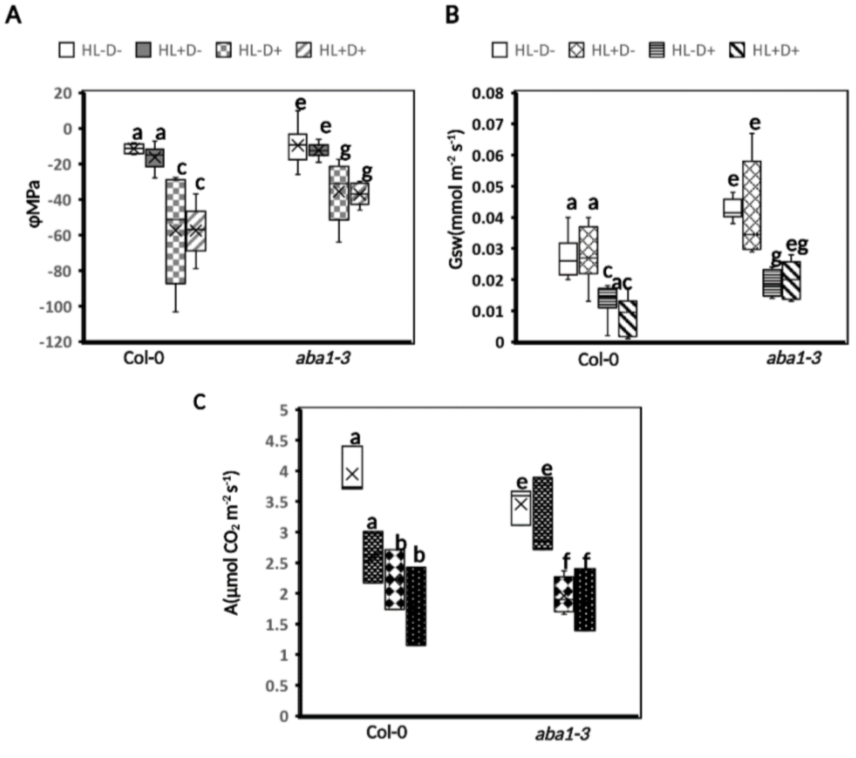

Figure S8. Water, conductance, and assimilation responses were triggered by D but HL treatment had little to no effect on these responses after subsequent D stress, related to Figure 7

(A) Relative water potential, (B) stomatal conductance, and (C) carbon assimilation responses were altered by D independent of HL pre-treatments. Measurements were taken at the end of the subjective drought period for all four treatments. The data are a representation of the second biological replicate with mean values. Statistical significance analysed by one-way ANOVA test and post-hoc Tukey's HSD test.

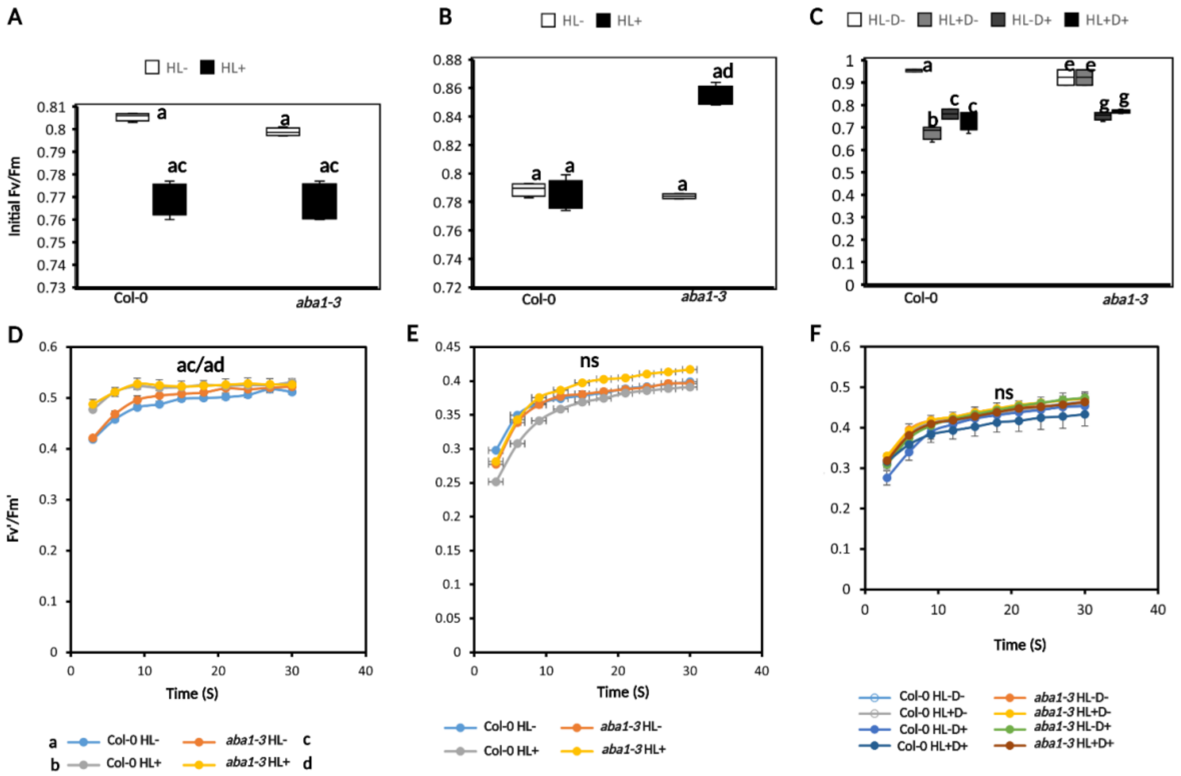

Figure S9. Photosynthetic initiation was not affected by D and HL stress, related to Figure 8

(A) Intial Fv/Fm measurement after HL stress. (B) Intial Fv/Fm after recovery. (C) Intial Fv/Fm after D stress. (D) Photosynthesis induction (Fv'/Fm') over 30 seconds after HL stress. (E) Photosynthesis induction after recovery. (F) Photosynthesis induction after D stress. Data is a representation of second biological replicate with mean values. Statistical significance analysed by one-way ANOVA test and post-hoc Tukey's HSD test.

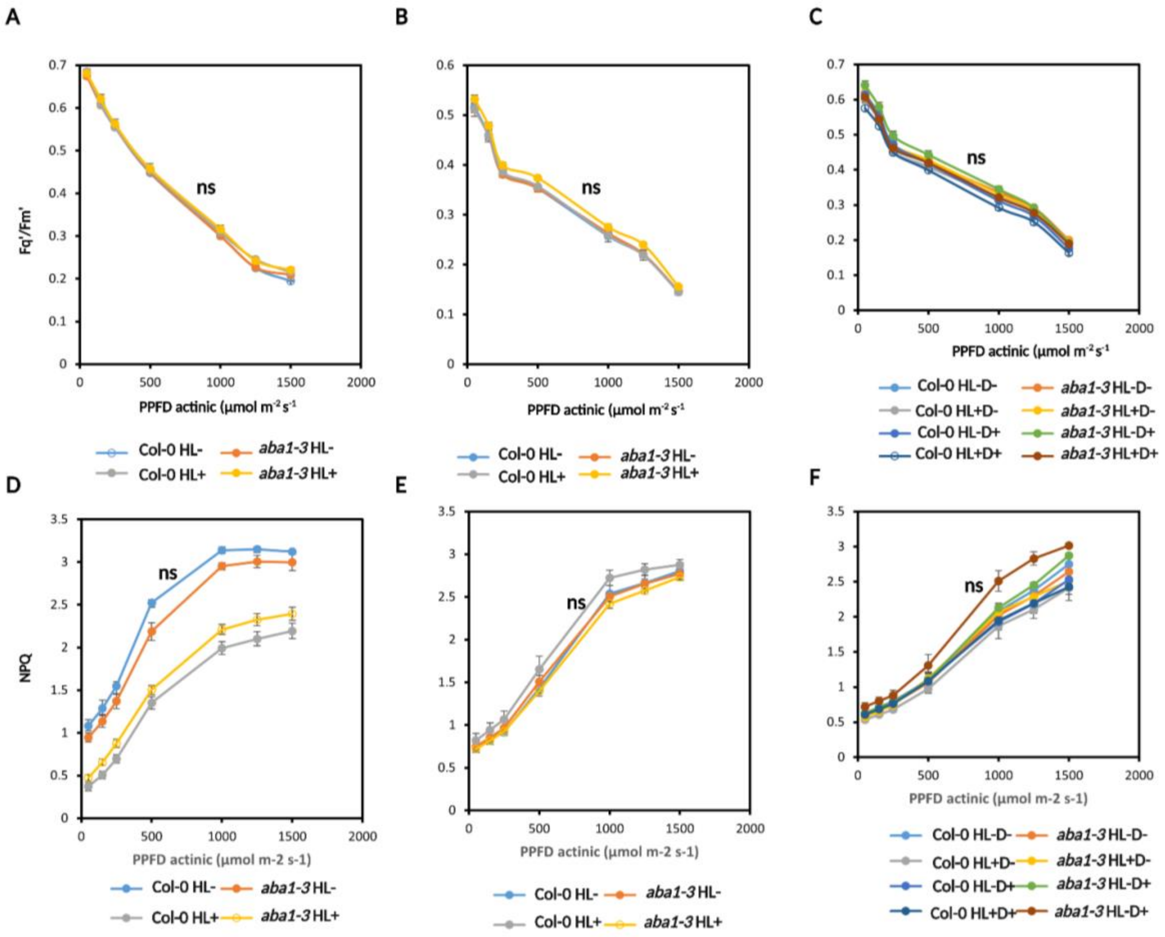

Figure S10. Photosynthesis performance was not affected in response to D and HL, related to Figure 9
(A) CF images of WT rosette after HL stress at first pixel measurements on Fo, Fm, F', Fm' , Fm/Fm'-1, and Fq'/Fm'. (B) Photosynthesis operating efficency measured by CF imaging using a light response curve of actinic light between 50-1500 µmol m-2 s-1 after HL stress. **(**C) Photosynthesis operating efficency measured after recovery stress. (D) Photosynthesis operating efficency measured after D stress. (E) NPQ measured using a light response curve of actinic light between 50-1500 µmol m-2 s-1 after HL stress. (F) NPQ measured after recovery. (G) NPQ measured after D stress. Data is a representation of second biological replicate with mean values. Statistical significance analysed by one-way ANOVA test and post-hoc Tukey's HSD test. ns = not significant.

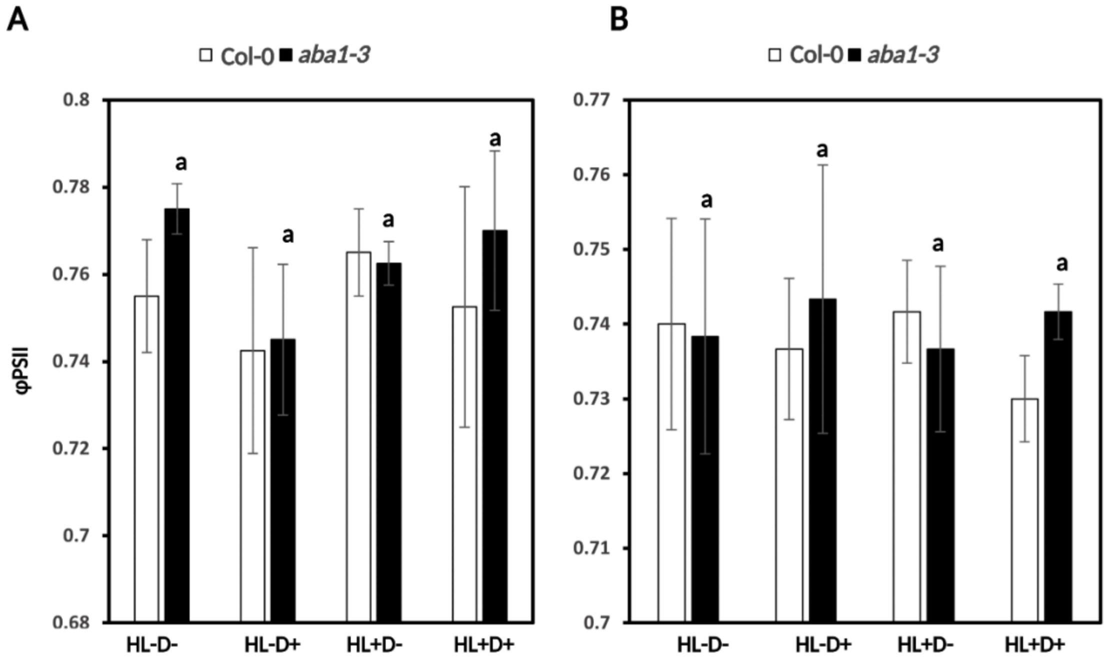

Figure S11: Quantum yield of fluorescence measured after D stress, related to Figure 9
(A-B) Data is a representation of two replicates. Statistical significance analysed by one-way ANOVA test and post-hoc Tukey's HSD test.

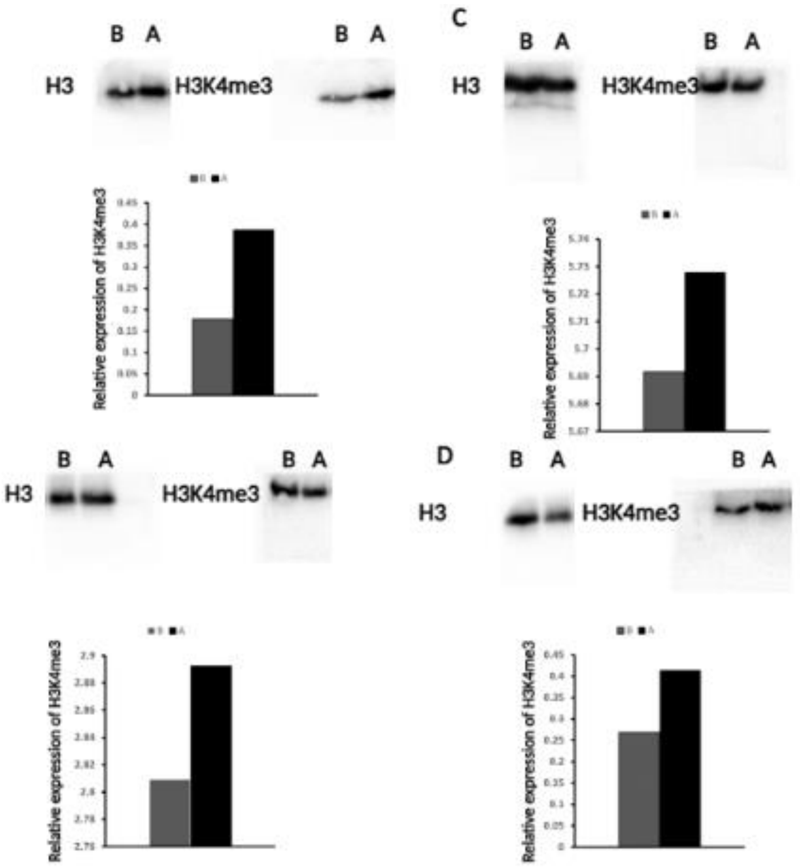

Figure S12. H3K4me3 increased in response to D and HL, related to Figure 10
(A-B) Immunoblot of WT plants stressed with D at 3-week-old. Histone extracts were probed with H3K4me3 specific antibodies, and H3 was used as loading control. Relative expression obtained from two independent replicate with densitometric analysis. (C-D) Immunoblot of WT plants stressed with HL at 3-week-old. Relative expression obtained from two independent replicate.

Table S1. Primers used in this study

qPCR	Forward	Reverse	Source
Act8	AAACCCGCTCTCGCTCTTAC	CTGAGTTTGAAACGCGGATT	This study
UBQ10	GGTTTGTGTTTTGGGGCCTTG	CGAAGCGATGATAAAGAAGAAGTTCG	This study
RD29A	CCGGAATCTGACGGCCGTTTA	CCGTCGGCACATTCTGTCGAT	Lee et al., 2016
ELIP1	TCGCAAGATCAACACCAACAA	AGGTTAGGGAAGCTACCGGC	This study
AREB1	AACAGGCTTACACCGTGGAG	CTTTGGACCTCCTTGCAGAA	Roca Paixao et al., 2019
DREB2	GACCTAAATGGCGACGATGT	TCGAGCTGAAACGGAGGTAT	Sakuma et al., 2006
ABA1	GATGCAGCCAAATATGGGTCAAGG	GCCATTGCATGGATAATAGCGACTC	This study
NCED3	GGTGGTTTACGACAAGAACAA	CAGAAGCAATCTGGAGCATCAA	This study
